# The utility of FDG-PET/CT imaging in the evaluation of multicentric reticulohistiocytosis: A case report: Erratum

**DOI:** 10.1097/MD.0000000000012617

**Published:** 2018-09-21

In the article, “The utility of FDG-PET/CT imaging in the evaluation of multicentric reticulohistiocytosis: A case report”,^[1]^ which appeared in Volume 97, Issue 33 of *Medicine*, Eosinophil and Myglobin were shifted incorrectly in Table 1. The corrected table appears below.

**Table 1:**
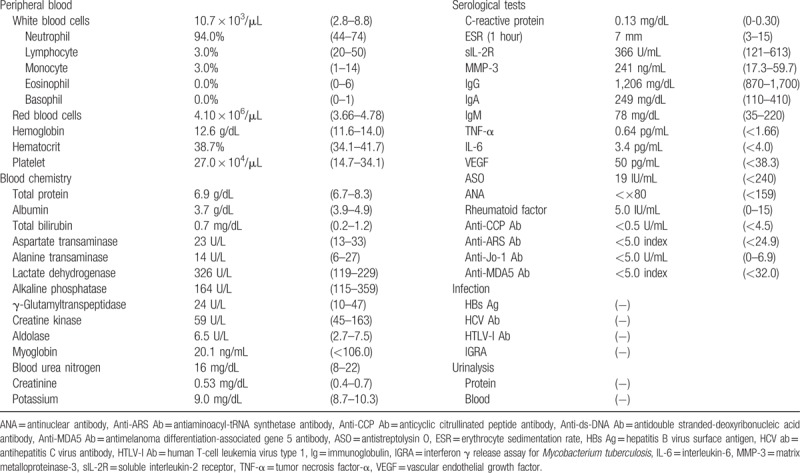
Laboratory findings on admission.

The caption for Figure 2 should appear as: X-ray of the hand. Marked erosion with punched-out resorptive lesion and dilatation of joint cleft, on the distal interphalangeal joint, were seen.

In addition, bone erosion and narrowing of the joint cleft were seen on carpometacarpal joint.
